# Simultaneous Quantification of Ivacaftor, Tezacaftor, and Elexacaftor in Cystic Fibrosis Patients’ Plasma by a Novel LC–MS/MS Method

**DOI:** 10.3390/biomedicines11020628

**Published:** 2023-02-20

**Authors:** Federica Pigliasco, Alessia Cafaro, Manuela Stella, Giammarco Baiardi, Sebastiano Barco, Nicoletta Pedemonte, Claudia D’Orsi, Federico Cresta, Rosaria Casciaro, Carlo Castellani, Maria Grazia Calevo, Francesca Mattioli, Giuliana Cangemi

**Affiliations:** 1 Department of Neurosciences, Rehabilitation, Ophthalmology, Genetics, Maternal and Child Health (DINOGMI), University of Genoa, 16132 Genoa, Italy; 2 Chromatography and Mass Spectrometry Section, Central Laboratory of Analysis, IRCCS Istituto Giannina Gaslini, 16147 Genoa, Italy; 3 Pharmacology & Toxicology Unit, Department of Internal Medicine, University of Genoa, Viale Benedetto XV 2, 16132 Genoa, Italy; 4 Clinical Pharmacology Unit, EO Ospedali Galliera, Mura Delle Cappuccine, 14, 16128 Genoa, Italy; 5 UOC Genetica Medica, IRCCS Istituto Giannina Gaslini, 16147 Genoa, Italy; 6 Cystic Fibrosis Center, IRCCS Istituto Giannina Gaslini, 16147 Genoa, Italy; 7 Epidemiology and Biostatistics Unit, Scientific Directorate, IRCCS Istituto Giannina Gaslini, 16147 Genova, Italy

**Keywords:** ivacaftor, tezacaftor, elexacaftor, CFTR, therapeutic drug monitoring, LC–MS/MS

## Abstract

The new breakthrough cystic fibrosis (CF) drug combination of ivacaftor (IVA), tezacaftor (TEZ), and elexacaftor (ELX), namely “caftor” drugs, directly modulates the activity and trafficking of the defective CF transmembrane conductance regulator protein (CFTR) underlying the CF disease. The role of therapeutic drug monitoring (TDM) of caftor drugs in clinical settings has recently been established. The availability of reliable and robust analytical methods for the quantification of IVA, TEZ, and ELX is essential to support dose–concentration–effect studies. We have developed and validated a new liquid chromatography–tandem mass spectrometry (LC–MS/MS) for the rapid and simultaneous quantification of IVA, TEZ, and ELX from the plasma of CF patients. The method was based on a rapid extraction protocol from 50 μL human plasma and separation on a reversed-phase C-18 HPLC column after the addition of deuterated internal standards. Accurate analyte quantification using multiple reaction monitoring (MRM) detection was then obtained using a Thermofisher Quantiva triple-quadrupole MS coupled to an Ultimate 3000 UHPLC. The method has been validated following international (EMA) guidelines for bioanalytical method validation and has been tested on plasma samples from 62 CF patients treated with the three-drug combination IVA/TEZ/ELX, marketed as Kaftrio^®^ or Trikafta^®^, in steady-state condition. The assay was linear over wide concentration ranges (0.008–12 mg/L) in plasma for IVA, TEZ, and ELX, suitable for a broad range of plasma concentrations, and accurate and reproducible in the absence of matrix effects. The stability of analytes for at least 30 days at room temperature could allow for cost-effective shipment and storage. On the same day of sample collection, a sweat test was evaluated for 26 associated patients’ samples, FEV1 (%) for 58, and BMI was calculated for 62. However, Spearman correlation showed no correlation between C_through_ plasma concentrations of analytes (IVA, TEZ, ELX) and sweat test, FEV1 (%), or BMI. Our method proved to be suitable for TDM and could be helpful in assessing dose–concentration–response correlations in larger studies.

## 1. Introduction

Cystic fibrosis (CF) is an autosomal recessive disease, caused by sequence variations in the gene encoding for the CF transmembrane regulator (CFTR), a channel protein that regulates chloride and bicarbonate transport [1]. The impaired function of CFTR results in a systemic disease affecting the upper and lower airways, liver, pancreas, and the gastrointestinal and reproductive tracts. Lung disease, with a pathogenic cascade of dehydrated airway surface liquid, impaired mucociliary clearance, chronic bacterial infections, disseminated bronchiectasis, and ultimately organ failure, is the major contributor to CF morbidity and mortality. Standard treatment includes symptomatic therapies such as pancreatic enzyme replacement, respiratory physiotherapy, airway hydrators, and an aggressive antibiotic pressure [2,3,4,5,6]. CFTR modulators, also named “caftor”, represent a recent therapeutic approach aimed at restoring defective CFTR protein function in patients carrying rescuable mutations. A novel drug named Kaftrio^®^ in Europe and Trikafta^®^ in the US (Vertex Pharmaceuticals Inc.) combines three pharmacological agents: ivacaftor (IVA), tezacaftor (TEZ), and elexacaftor (ELX). It is used in patients aged 6 years and older with at least one F508del mutation, the most frequent disease-causing CF mutation [4,7,8,9].

TEZ and ELX are CFTR correctors, small molecules that improve the folding and maturation of F508del mutated CFTR; IVA is a CFTR potentiator and increases the channel open probability [10]. TEZ, ELX, and IVA clinical trials have shown unprecedented positive outcomes in patients bearing one or two copies of the F508del allele [11,12,13], but a non-negligible inter-subject variability in response to treatment has also been reported [4,14]. Noticeably, caftor drugs are metabolized by cytochrome P450 subtypes CYP3A4 and CYP3A5, which are inhibited by several drugs commonly co-administered in CF patients (ketoconazole, itraconazole, posaconazole, voriconazole, clarithromycin, fluconazole, erythromycin, and verapamil) [15]. Plasma quantitation of drugs is a very useful clinical tool for the therapeutic optimization of critical drugs through adjustment of drug exposure by therapeutic drug monitoring (TDM). Drugs are suitable for TDM when they are characterized by significant between-subject variability (BSV) in drug exposure and by a plasma concentration–response and/or toxicity–toxicity relationship, which define concentration ranges associated with optimal efficacy and minimal toxicity [16]. TDM might be therefore useful to optimize treatments with CFTR modulators [16]. Reliable analytical methods for the plasma-level determination of IVA, TEZ, and ELX are essential to collect dose–concentration–response data [4].

In this paper, we report on a new analytical method based on reversed-phase liquid chromatography coupled to Thermofisher Quantiva triple-quadrupole MS (LC–MS/MS) for the determination of IVA, TEZ, and ELX on plasma samples. The method has been validated according to current international guidelines and successfully applied to clinical samples obtained from CF patients under steady-state treatment with Kaftrio^®^ [17,18].

## 2. Materials and Methods

### 2.1. Chemicals and Reagents

Ivacaftor (ref. I940600), tezacaftor (ref. T321510), and elexacaftor (ref. E201545) were purchased from Toronto Research Chemicals (North York, ON, Canada). Ivacaftor d-9 (ref. I940603), tezacaftor d-9 (T321512), and elexacaftor d-3 (E201547) were purchased from Spectra 2000 (Rome, Italy).

High-performance liquid chromatography (HPLC)-grade methanol and acetonitrile (ACN) and LC–MS/MS-grade formic acid (ref. 607001000) were purchased from Sigma-Aldrich Srl. (Milan, Italy). All reagents had 98% purity.

All solutions were prepared with HPLC-grade water obtained from a Milli-Q Plus water purification system (18.2 MΩ•cm, 25 °C, TOCs < 5 ppb). HPLC mobile phases were filtered by using Millipore membrane filters (0.45 µm) (Millipore, Vimodrone MI, Italy).

### 2.2. Calibration Curve, Quality Control, and Stock Solution Preparation

IVA, TEZ, and ELX were dissolved in DMSO to obtain stock solutions at 2.6 mg/mL, 1.6 mg/mL, and 2.5 mg/mL, respectively. Stock solutions of ivacaftor d-9 (IS) (1 mg/mL), tezacaftor d-9 (0.5 mg/mL), and elexacaftor d-3 (0.5 mg/mL) were prepared by dissolving any substance in DMSO. Calibrators and quality controls (QC) were obtained by spiking a pool of blank plasma with analytes from different batches of working solutions of IVA, TEZ, and ELX. The 9-point calibration curve, ranging from 0.008 to 12 mg/L (0.008, 0.020, 0.049, 0.123, 0.307, 0.768, 1.920, 4.8, 12 mg/L), included the lower limit of quantitation (LLOQ). QC samples were prepared at the following concentrations: 0.36 mg/L (QC low), 6 mg/L (QC medium), and 9 mg/L (QC high).

### 2.3. Human Samples

The study was approved by our Internal Review Board (0037117/22-01/12/2022) and written informed consent was obtained from all patients or legal representatives at the time of admission to use clinical data for research purposes, following the IRCCS Istituto Giannina Gaslini, Genoa, Italy, Privacy Policy.

Blank samples for method validation purposes were obtained from healthy adult volunteers who were not being treated with IVA, TEZ, and ELX. The suitability of the developed method was tested on 62 leftover plasma samples collected for routine analyses from CF patients who received treatment with Kaftrio^®^ according to the summary of product characteristics (SmPC) [15]: two tablets containing 75 mg IVA/50 mg, TEZ/100 mg, and ELX in the morning, and one IVA/150 mg tablet in the evening, approximately 12 h apart.

Patient samples were obtained from 10 children (12–15 years), 16 young adults (15–25 years), and 36 adults (>25 years), followed at the Giannina Gaslini Children’s Hospital. Patients’ demographics characteristics are shown in Table 1. Blood samples to measure caftor concentrations were obtained during routine visits, immediately before the daily administration of the first tablet in the morning (C_trough_) and in conditions of plasma drug concentration stability (steady state), as all patients had been on therapy for at least 12 weeks, according to SmPC [15]. Plasma was separated from peripheral blood collected in tubes with K3-EDTA anticoagulant by centrifuging at 4000× *g* for 5 min and stored at −20 °C until analyzed. At sample collection, a sweat test was performed in 26 patients, forced expiratory volume in the first second (FEV1) in 58 patients, and body mass index (BMI), calculated by dividing weight in kilograms by height in meters squared (Kg/m^2^), in 62 patients.

The pulmonary function of CF patients is routinely assessed by measuring the spirometric parameter FEV1 as it has been shown to be a good predictor of mortality in these patients [19]. The percentage of predicted forced expired volume in one second (FEV1pp) values ≥90% are rated normal, values between 89 and 70% normal/moderate, values between 40 and 69% moderate, and FEV1pp values below 40% are considered severe [20].

The gold standard for the diagnosis of CF is the sweat test, which assesses the amount of chloride in the patient’s sweat. Sweat chloride values (CLsw) ≥ 60 mEq/L are diagnostic of CF, those between 30 and 59 mEq/L are considered borderline, and values lower than 30 mEq/L are normal [21,22]. At the same time as blood sampling was performed to measure caftor concentrations, the sweat test was performed in 26 patients, FEV1 (%) in 58 patients, and biometric variables, weight, height, and BMI were calculated in 62.

### 2.4. Sample Preparation

A 50 μL aliquot of plasma (calibrators, QCs, and patient samples) was protein-precipitated with 200 μL methanol, after the addition of 10 µL IS working solution (0.5 mg/mL). After vortexing, samples were centrifuged at 14000× *g* for 5 min at 4 °C.

### 2.5. Chromatographic Conditions

Gradient separation chromatography was carried out on Ultimate 3000 UHPLC Dual-Gradient Pumps (Thermo Fisher Scientific, Milan, Italy) out using Accucore Polar Premium (50 mm × 2.1 mm, i.d. 2.6 µm, Thermo Scientific, Milan, Italy), with mobile phase A consisting of 0.1% formic acid (FA) in water and mobile phase B consisting of 0.1% formic acid in acetonitrile. No pre-column was used. The gradient started at 5% of phase B; after 2 min, it was programmed to reach 100% in 1 min at a flow rate of 500 µL/min. After maintenance of these conditions for 1 min, the column was washed with 5% B for 2 min, for a total run time of 5 min. The column temperature was maintained at 50 °C and the injection volume was 5 µL.

### 2.6. MS/MS Conditions

Detection was carried out using a TSQ Quantiva triple-quadrupole system (Thermo Fisher Scientific, Milan, Italy) equipped with an electrospray ionization source (ESI) operating in the positive ion mode (spray voltage at 3500 V). Nitrogen was used as the nebulizer and auxiliary gas, set at 50 and 15 arbitrary units, respectively; vaporizer and capillary temperature setting for both was 350 °C and argon was used as a collision gas at a pressure of 1.5 mTorr.

The following specific transitions were detected using multiple reaction monitoring (MRM): 393.3→144.1; 319.1 for IVA, 521.3→387.0; 503.2 for TEZ; and 598.2→328.9; 423.3 for ELX. Deuterated IS were detected using the following transitions: 402.5→328.1 for ivacaftor d-9; 601.3→502.2 for elexacaftor d-3; 525.2→391.1 for tezacaftor d-4.

### 2.7. Method Validation

#### 2.7.1. Selectivity

Selectivity was tested by analyzing samples from six healthy volunteers not assuming drugs. A blank human sample, spiked with analytes at the LLOQ, and a sample spiked with IS were processed and analyzed using the same method for each batch. In accordance with EMA guidelines, the absence of interfering components was considered as acceptable when the signal was less than 20% of the LLOQ for IVA, TEZ, and ELX and less than 5% for their IS.

#### 2.7.2. Carry-Over

The presence of carry-over was tested by injecting blank samples in triplicate after the highest calibration standard. According to EMA guidelines, carry-over was considered acceptable if the signal in the blank sample following the higher standard was less than 20% of the LLOQ and 5% for the IS.

#### 2.7.3. Matrix Effects and Extraction Recovery

Matrix effect and extraction recovery for IVA, TEZ, and ELX and IS were assessed at two different levels (corresponding to the low and high QC) analyzed in triplicate on 6 lots of blank matrix samples from individual donors. Matrix effects were determined by comparing the peak area of the analytes spiked after extraction to the peak area of pure solution at the same concentration according to B.K. Matuszewski et al. [23]. Extraction recovery was investigated by comparing the peak area of IVA, TEZ, and ELX spiked before extraction to the peak area of IVA, TEZ, and ELX after extraction.

#### 2.7.4. Linearity

Linearity was evaluated by analyzing the calibration curve three times on three non-consecutive days. The peak area ratio of analyte/IS vs. the analyte concentration of each calibration standard was fitted by using a 1/x weighting factor. A weighted (1/*x*) quadratic regression model was used because the absolute variation is larger for higher concentrations and the data at the high end of the calibration curve tend to dominate the calculation of the linear regression, often resulting in excessive error at the bottom of the curve [24].

The statistics of the mean calibration curves were the following: Y= 1.5 × 10^3^ + 4.8 × 10^5^ X − 2.7 × 10^3^ X^2^ with R^2^ = 0.9998 for IVA, Y = 9.3 × 10^−3^ + 5.2 × X + 1.1 × 10^−1^ X^2^ with R^2^ = 0.9991 for TEZ, and Y = −2.0 × 10^−3^ + 1.6 × X + 2.5 × 10^−2^ X^2^ with R^2^ = 0.9995 for ELX. The calibration curves were validated in the concentration range 0.008–12 mg/L. The acceptance criteria for the back-calculated concentrations of calibration standards were 15% of the theoretical value, except for the LLOQ (± 20%).

#### 2.7.5. Precision, Accuracy, and LLOQ

Within-run and between-run precision and accuracy were estimated by testing the four-level QC samples five times on three separate days. Accuracy was expressed as the mean relative error (expressed as a percentage) and precision as the coefficient of variation (CV%). Acceptable ranges were considered within 85–115% and ≤15% of the nominal concentrations for accuracy and precision, respectively. The LLOQ was defined as the lowest concentration that could be measured with precision ≤20% and accuracy within 80–120% of the nominal concentration. Moreover, the LLOQ should have a signal to noise ratio >5. Dilution integrity was assessed by diluting 2- and 5-fold (*v/v*) the highest calibration standard with blank matrix and analyzing the diluted sample fivefold.

#### 2.7.6. Stability

Stability was assessed by analyzing three replicates of QC low and QC high in plasma after storage at RT for 4 weeks, and at −20 °C for 4 weeks, respectively.

Stability was evaluated on five real samples by analyzing them after storage at RT for 4 weeks, at +4 °C for 4 weeks, and at −80 °C for 4 weeks, respectively.

Stability was considered acceptable if the percentage difference, calculated as the ratio between the concentration measured at each sampling point and the initial concentration, was lower than 15%, in compliance with EMA guidelines [17].

### 2.8. Statistical Analyses

Descriptive statistics were generated for the whole cohort and data were expressed as mean and standard deviation for continuous variables. Median value and range were calculated and reported, as were absolute or relative frequencies for categorical variables. Spearman correlation coefficients were used to analyze relations between C_trough_ of IVA, TEZ, and ELX and the values of the sweat test, FEV1 (%), and BMI. A *p*-value less than 0.05 was considered statistically significant, and all *p*-values were based upon two-tailed tests. Statistical analyses were performed using the Statistical Package for the Social Sciences (SPSS) for Windows (SPSS Inc., Chicago, IL, USA).

## 3. Results

### 3.1. Method Development

To optimize the extraction conditions, we tested methanol and acetonitrile with or without the addition of formic acid (0.1%).

The extraction procedure that gave the best results in terms of extraction recovery (ER) is described in the Section 2.

The HPLC column chosen for the chromatographic separation allowed good separation efficiency and a good peak shape. Retention times for IVA and ELX were 2.78 min (±0.10) and that for TEZ was 2.41 min (±0.10), respectively.

### 3.2. Method Validation

The method was validated following EMA guidelines [17] and successfully applied to clinical samples obtained from CF patients under steady-state treatment with Kaftrio^®^. The figures reported showed an excellent analytical method.

**Figure 1:**
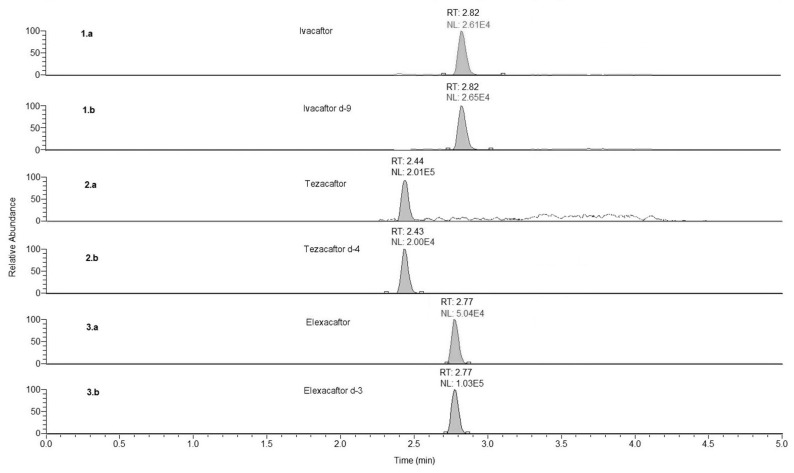
Chromatograms obtained: a calibrator at the LLOQ in plasma (panel 1.a, ivacaftor; panel 2.a, tezacafor, panel 3.a, elexacaftor); deuterated internal standards (panel 1.b, ivacaftor-d9, panel 2.b, tezacafor-d4, panel 3.b, elexacaftor-d3). RT, retention time. NL, normalized level.

**Figure 2:**
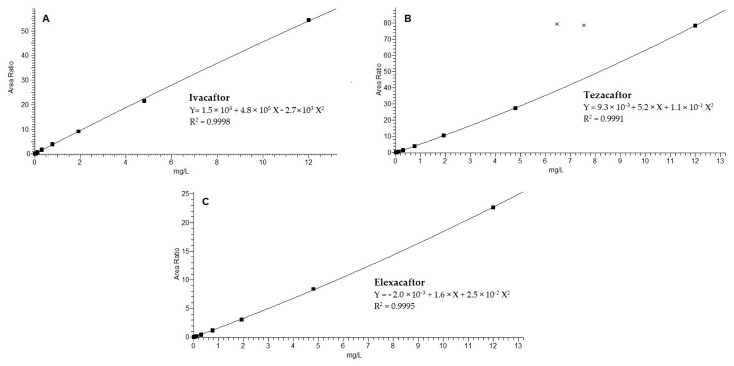
The mean calibration curves in plasma (9-point calibration curve) of ivacaftor (**A**), tezacafor (**B**), and elexacaftor (**C**), ranging from 0.008 to 12 mg/L. Area Ratio, ratio between area standard and IS.

At the LC–MS/MS conditions described in Section 2.5 and Section 2.6, no interfering peaks could be detected. Carry-over could be considered negligible. The LLOQ obtained was 0.008 mg/L for the three drugs. Representative chromatograms obtained are shown in Figure 1. A linear relationship between the analytes’ peak area and the corresponding concentration was achieved (with R^2^ = 0.99) in the described concentration range (Figure 2) and the back-calculated concentration values for the three drugs were not significantly different from the nominal value (±15%). In compliance with EMA guidelines [17], CV% was < 12% for the nine calibrators and < 19% for the LLOQ, intra- and inter-assay precision, and accuracy (Table 2).

Recovery and matrix effect results were acceptable according to the EMA guidelines [17].

Dilution integrity met the acceptance criteria for accuracy (±15% of the nominal value). Analyses carried out to assess the matrix effect and IS-normalized matrix effect yielded results within acceptable ranges (in the range of 8–12%).

Extraction recovery tests results were 86% for IVA, 97% for TEZ, and 99% for ELX with CV% <15%. Short-term and long-term stability tests demonstrated that all analytes were stable in plasma at RT, +4 °C, and −80 °C after 4 weeks (Figures S1–S3).

### 3.3. Analysis of Patients’ Samples

The novel method was applied on 62 samples derived from patients under treatment with the combination drug. Plasma levels obtained (mean ± SD) were 3.14 ± 1.35 mg/L for TEZ, 5.22 ± 2.10 mg/L for ELX, and 0.85 ± 0.39 mg/L for IVA. No significant differences in plasma concentrations were observed among the three groups (children, young adults, adults). Data are shown in Table 3. As suggested by the EMA guidelines, all samples were tested in two different analytical runs to evaluate the incurred sample reanalysis precision. The results (inter-day RSD = 10%) showed acceptable reproducibility.

CLsw values obtained were ≥ 60 mEq/L in 12 patients, 30 mEq/L- 59 mEq/L in 11 patients, and ≤ 30 mEq/L in 3 patients. The measured values of FEV1pp on 58 samples were as follows: 27 patients had FEV1pp ≥ 90%, 14 patients had FEV1pp between 89 and 70%, 13 patients had FEV1pp between 40 and 69%, and 4 patients had FEV1pp below. 40%. BMI was 17.0–18.5 in 7 patients, 18.6–24.4 in 44 patients, and 25.0–28.5 in 11 patients.

### 3.4. Correlation Analyses

Since key pharmacokinetic parameters (PKs) at steady state (i.e., C_max_, the highest concentration in the blood or AUC_0–24_, the area under the concentration–time curve at steady state over 24 h) for IVA, TEZ, and ELX seemed to be comparable across the whole CF patient population aged >12 years [15], we evaluated the Spearman correlation between the C_through_ of analytes (IVA, TEZ, ELX) and the sweat test, FEV1, and BMI, respectively. No statistical correlation could presently be demonstrated; scatter plots are shown in the Supplementary Materials (Figures S4–S12).

## 4. Discussion

In this paper, we show a novel LC–MS/MS method for the fast and specific quantification of IVA, TEZ, and ELX with high accuracy and precision over a wide range of concentrations starting from low (50 μL) volumes of plasma. The use of deuterated internal standards of the three drugs guarantees sufficient robustness, allowing its application for TDM. Candidate drugs for TDM have a significant interindividual PK variability profile, poorly predictable from individual patients’ characteristics [25,26].

CF is associated with such pathological alterations influencing significantly the PK profiles of several drugs [6]. Furthermore, a number of drug interactions (DDIs) could alter the exposure of caftors due to their CYP3A4- and CYP3A5-dependent metabolism. Many commonly co-administered drugs in CF patients can inhibit CYP3A (e.g., azole antifungals) or are metabolized by CYP3A (e.g., antibiotics, steroids) [16], suggesting their potential influence on pharmacokinetic variability in caftor exposure. The caftor dose–response relationships with the usual CF disease parameters (i.e., sweat chloride concentration, FEV1, and BMI) show high variability in the treatment response, found even in patients with the same CFTR genotype and dosage regimen [14], suggesting that interindividual differences in PKs are likely to explain, at least in part, such inconsistency in drug responses [16].

The best time after dose (TAD) for blood collection, e.g., C_max_, C_through_, or AUC determination, for obtaining clinically relevant TDM information remains to be formally determined for caftors. However, calculation of the AUC requires multiple blood draws for an entire dosing interval, which is not feasible in a routine clinical setting [16]. The C_through_ correlation with FEV1, the sweat test, and BMI was therefore selected to be investigated in our patients’ setting.

Few papers have been previously published on analytical methods for the quantification of CF modulators [27,28,29,30,31,32]. Very few of them report on the simultaneous quantification of IVA, TEZ, and ELX [27,28,31]. To our knowledge, only one paper [28] showed robust validation LC–MS/MS protocols suitable for TDM of the three drugs using isotope-labelled internal standards. Our method is based on a simple sample preparation procedure combined with one-step chromatographic separation and could be easily transferred to other laboratories, as opposed to an SPE online method used by other authors [28]. In the literature, different organic agents such as ACN [29], ACN with 0.1% FA [31,32], methanol:ACN (1:5.25) [27], and methanol [28,30], with different volumes of plasma:organic phase ratios (from 1:2 to 1:5), were used and different plasma volumes (from 10 µL to 200 µL) were adopted. Liquid chromatography has been mainly coupled to triple quadrupole, [27,28,29,30,31,32,33], as opposed to HPLC–UV [32], since LC–MS/MS is considered nowadays as the gold standard due to its higher specificity in the quantification of small molecules.

Our method has been clinically validated by application to a relevant number of plasma samples obtained from CF patients under treatment with IVA, TEZ, and ELX at steady state.

Very few papers have reported the results of the measurement of IVA, TEZ, and ELX in the plasma of CF patients, and only in one of them [28] were they obtained from a relevant number of samples. Furthermore, in the present paper, for the first time, results of the plasma levels of CF patients obtained at C_trough_ were correlated with the clinical response. No statistically significant relation between C_trough_ drug levels and the usual CF parameters defining the clinical response could be demonstrated.

A considerable number of side effects have been reported in association with the use of Kaftrio^®^, including a significant increase in transaminases, bilirubin, creatinkinase, rashes, anxiety, depression, and suicidal ideation (REF). In the occurrence of such events, it may be necessary to decrease the daily dose or even discontinue Kaftrio^®^, and, if and once the event resolves, gradually reintroduce the modulator. The process of scaling down Kaftrio^®^ might be accompanied by a TDM protocol, which might help to identify the dosage that causes the side effect and contribute to the adjustment of the prescription. Quantification of the plasmatic levels of IVA, TEZ, and ELX will also prove useful to gauge adherence to treatment. Although modulators have been proven to be life-changing medicines in the vast majority of those taking them, there still patients who are poorly adherent to the therapeutic schedule [34], or even not taking the medication at all. Moreover, proper absorption requires that Kaftrio^®^ is taken together with a meal or snack containing fats, a recommendation that may be inadequately followed by patients. A TDM protocol for the simultaneous quantification of IVA, TEZ, and ELX from the plasma of CF patients could be a valuable tool in clinical practice to ascertain inadequate clinical responses due to insufficient drug exposure or imperfect treatment adherence.

## 5. Conclusions

We have shown the development and validation of an accurate and reproducible LC–MS/MS method from a very low amount (50 μL) of plasma for the simultaneous measurement of IVA, TEZ, and ELX. Although Spearman correlation showed no relation between the plasma C_trough_ concentrations of analytes (IVA, TEZ, ELX) and the sweat test, FEV1, or BMI, our method proved to be suitable for TDM and could be easily adopted by other clinical laboratories. Simultaneous TDM of IVA, TEZ, and ELX could be valuable in assessing the interindividual variability of the PK profile and dose–concentration–response correlations in larger studies.

## Figures and Tables

**Table 1:** Patients’ demographic characteristics. SD: standard deviation.

	Males (*n* = 28)		Females (*n* = 34)	
	Age (Yrs)	Weight (Kg)	Age (Yrs)	Weight (Kg)
Mean	30.4	66.8	30.4	53.5
SD	10.6	10.9	16.5	7.99
Median	32.4	66.6	23.9	52.5
Min–Max	13–51	38.0–84.0	12–75	40.5–79.8

**Table 2:** Results of intra-day and inter-day accuracy and reproducibility assays (n = 5). The quality controls’ concentrations were 0.008, 0.04, 0.41, 5 mg/L for LLOQ, QClow, QC medium, and QC high, respectively (CV% is the coefficient of variation percentage).

**INTER-DAY**									
	**Ivacaftor**			**Tezacaftor**			**Elexacaftor**		
	**Nominal value (mg/L)**	**CV%**	**Accuracy %**	**Nominal value (mg/L)**	**CV%**	**Accuracy %**	**Nominal value (mg/L)**	**CV%**	**Accuracy %**
**LLOQ**	0.008	13%	115%	0.008	13%	109%	0.008	5%	107%
**QC low**	0.04	11%	114%	0.04	10%	99%	0.04	4%	106%
**QC medium**	0.41	9%	91%	0.41	4%	98%	0.41	4%	98%
**QC high**	5	9%	92%	5	2%	92%	5	8%	107%
**INTRA-DAY**									
	**Ivacaftor**			**Tezacaftor**			**Elexacaftor**		
	**Nominal value (mg/L)**	**CV%**	**Accuracy %**	**Nominal value (mg/L)**	**CV%**	**Accuracy %**	**Nominal value (mg/L)**	**CV%**	**Accuracy %**
**LLOQ**	0.008	14%	115%	0.008	9%	105%	0.008	7%	105%
**QC low**	0.04	3%	110%	0.04	7%	104%	0.04	3%	99%
**QC medium**	0.41	8%	110%	0.41	5%	99%	0.41	4%	98%
**QC high**	5	9%	91%	5	2%	106%	5	3%	108%

**Table 3:** TEZ, ELX, and IVA plasma levels (mg/L) expressed as mean ± SD in children, young adults, and adults.

	TEZ (mg/L)	ELX (mg/L)	IVA (mg/L)
**Children**	2.68 ± 0.92	4.39 ± 1.59	0.71 ± 0.20
**Young Adults**	2.87 ± 1.38	4.70 ± 2.26	0.84 ± 0.52
**Adults**	3.40 ± 1.40	5.68 ± 2.07	0.90 ± 0.37

## Data Availability

The data presented in this study are available from the corresponding author upon special request.

## Supplementary Materials

The following supporting information can be downloaded at: https://www.mdpi.com/article/10.3390/biomedicines11020628/s1. Figure S1: Stability at room temperature of Tezacaftor, Elexacaftor and Ivacaftor, on five real samples (mg/L). Figure S2: Stability at +4 °C of Tezacaftor, Elexacaftor and Ivacaftor, on five real samples (mg/L). Figure S3: Stability at −80 °C of Tezacaftor, Elexacaftor and Ivacaftor, on five real samples (mg/L). Figure S4: The scatter plot between C_through_ of TEZ and sweat test. Figure S5: The scatter plot between C_through_ of TEZ and FEV1 (%). Figure S6: The scatter plot between C_through_ of TEZ and BMI. Figure S7: The scatter plot between C_through_ of ELX and sweat test. Figure S8: The scatter plot between C_through_ of ELX and FEV1 (%). Figure S9: The scatter plot between C_through_ of ELX and BMI. Figure S10: The scatter plot between C_through_ of IVA and sweat test. Figure S11: The scatter plot between C_through_ of IVA and FEV1 (%). Figure S12: The scatter plot between C_through_ of IVA and BMI.
