# Supplementary material for: Simultaneous Quantification of Ivacaftor, Tezacaftor, and Elexacaftor in Cystic Fibrosis Patients’ Plasma by a Novel LC–MS/MS Method
Source: Biomedicines. 2023 Feb 20;11(2):628. doi: 10.3390/biomedicines11020628 (PMC9953078; doi:10.3390/biomedicines11020628)

SUPPLEMENTARY MATERIALS

Supplementary Figure S1: Stability at room temperature (RT) of Tezacafor, Elexacaftor and Ivacaftor on five real samples (mg/L).

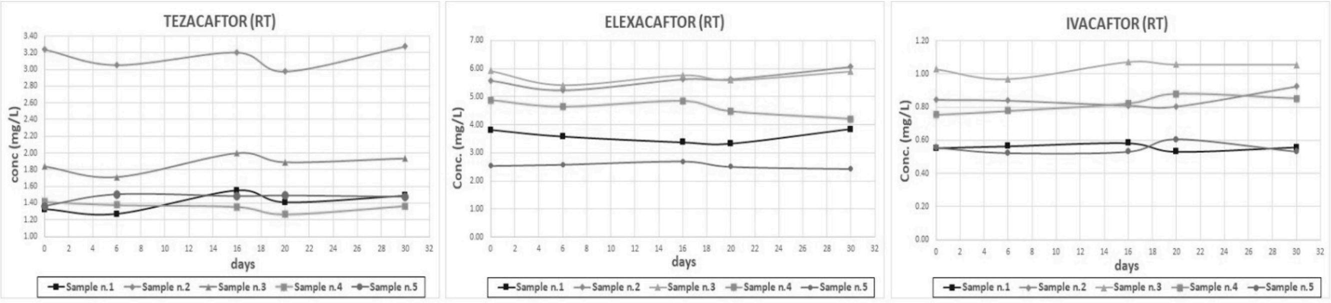

Supplementary Figure S2: Stability at + 4°C of Tezacafor, Elexacaftor and Ivacaftor on five real samples (mg/L).

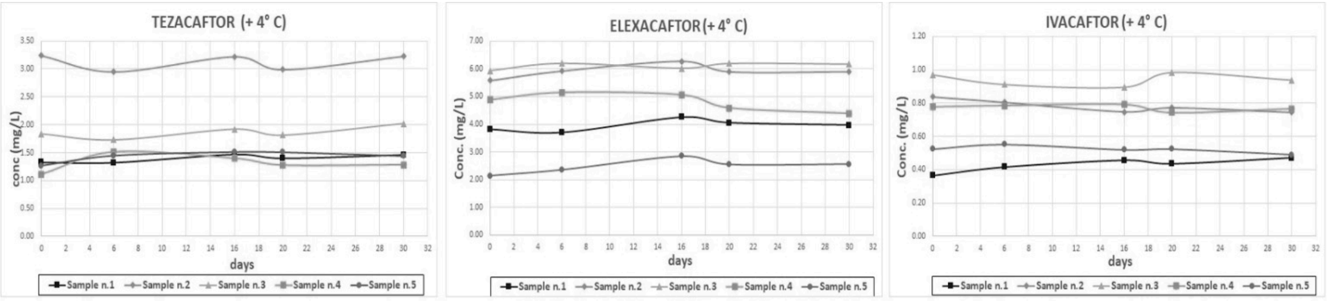

Supplementary Figure S3: Stability at -80°C of Tezacafor, Elexacaftor and Ivacaftor on five real samples (mg/L).

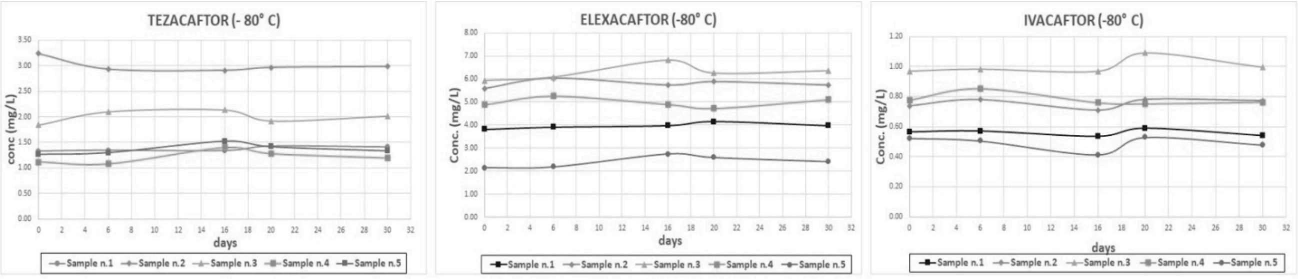

Supplementary Figure S4: The scatter plot between Cthrough of TEZ and sweat test.

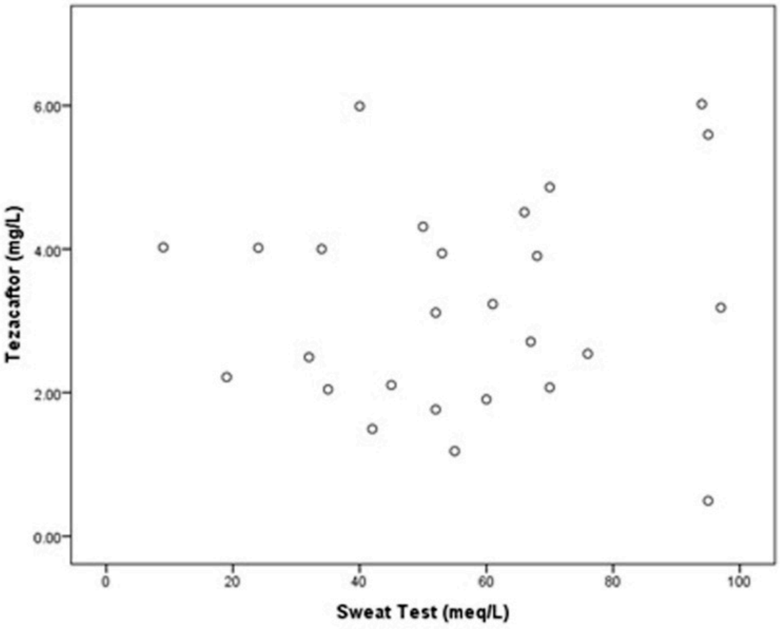

Supplementary Figure S5: The scatter plot between Cthrough of TEZ and FEV1 (%).

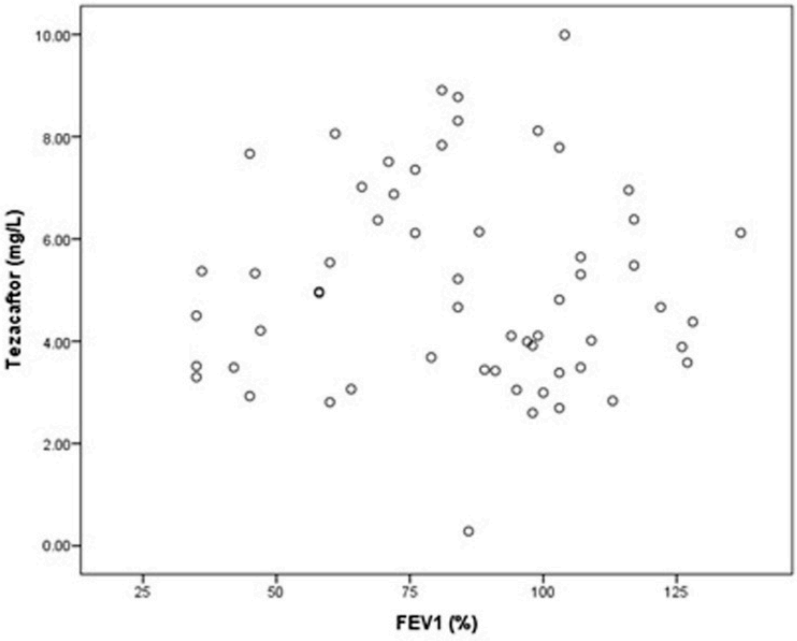

Supplementary Figure S6: The scatter plot between Cthrough of TEZ and BMI.

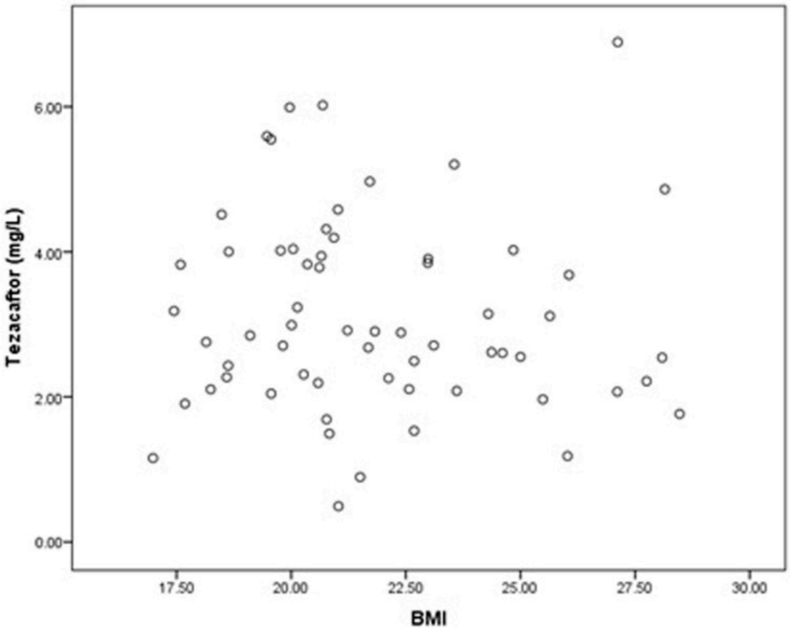

Supplementary Figure S7: The scatter plot between Cthrough of ELX and sweat test.

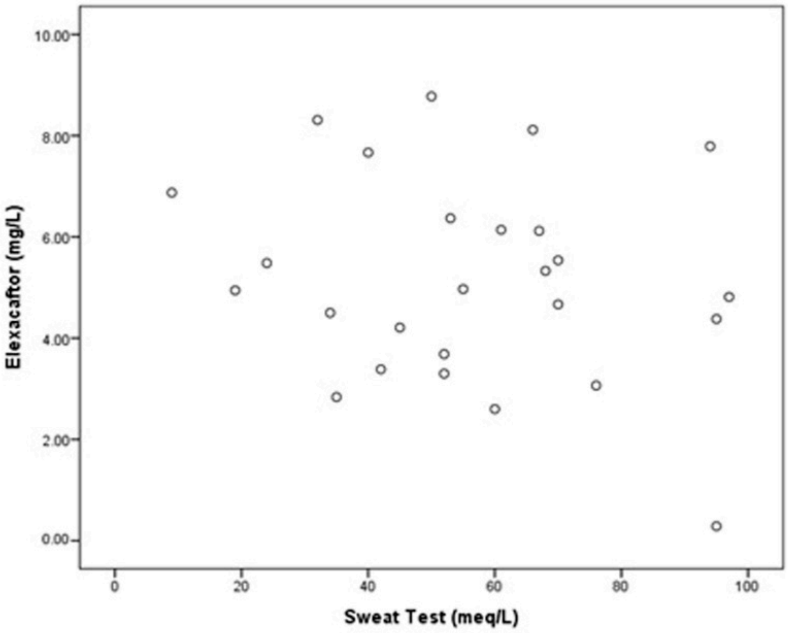

Supplementary Figure S8: The scatter plot between Cthrough of ELX and FEV1 (%).

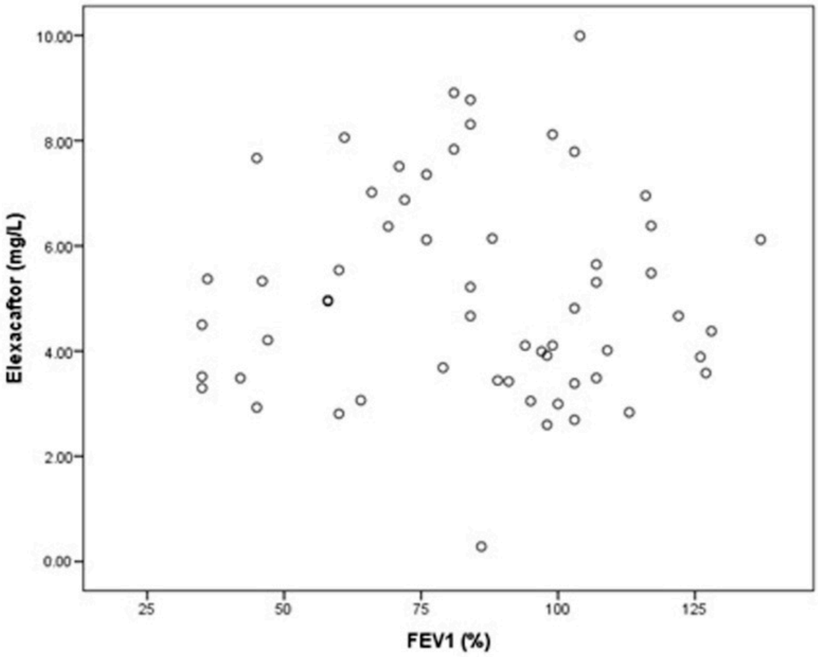

Supplementary Figure S9: The scatter plot between Cthrough of ELX and BMI.

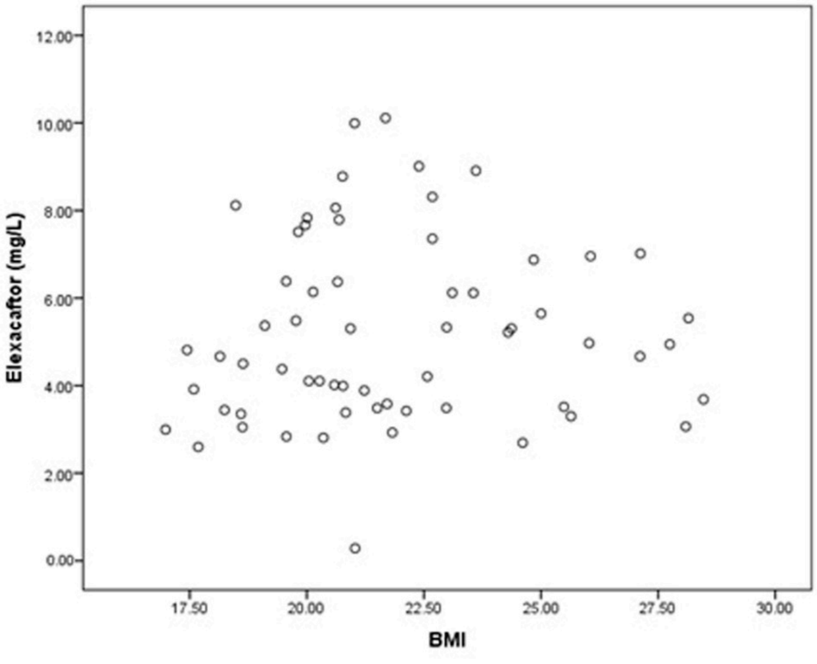

Supplementary Figure S10: The scatter plot between Cthrough of IVA and sweat test.

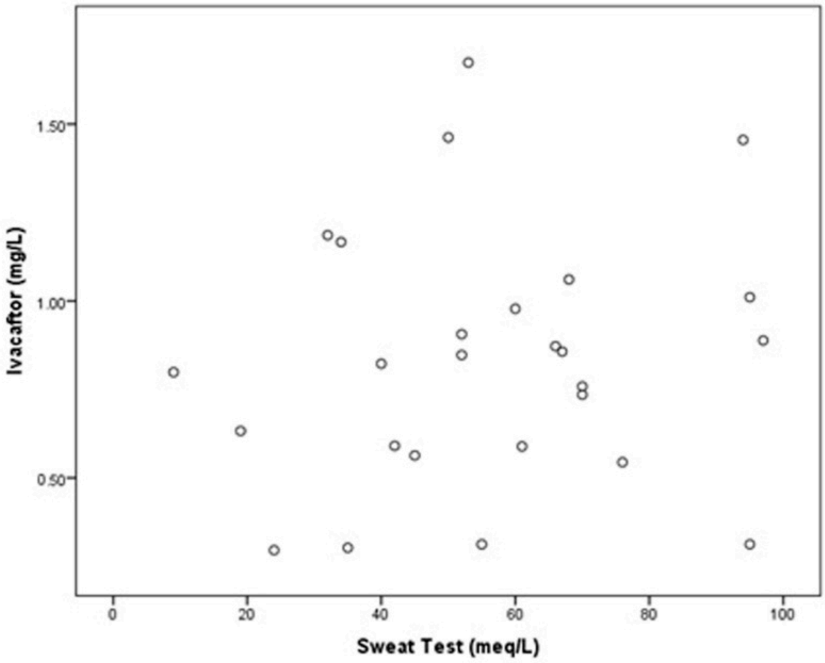

Supplementary Figure S11: The scatter plot between Cthrough of IVA and FEV1 (%).

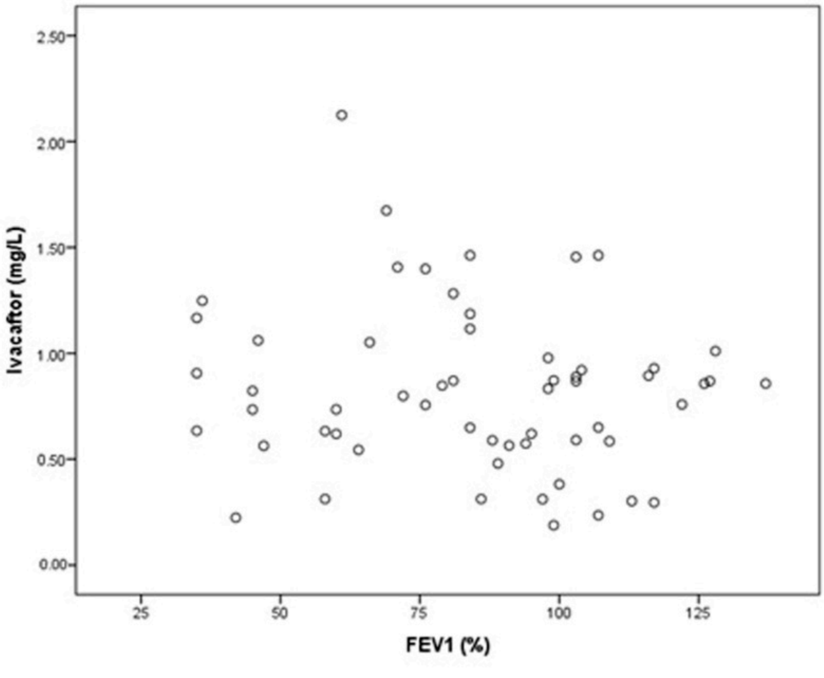

Supplementary Figure S12: The scatter plot between Cthrough of IVA and BMI.

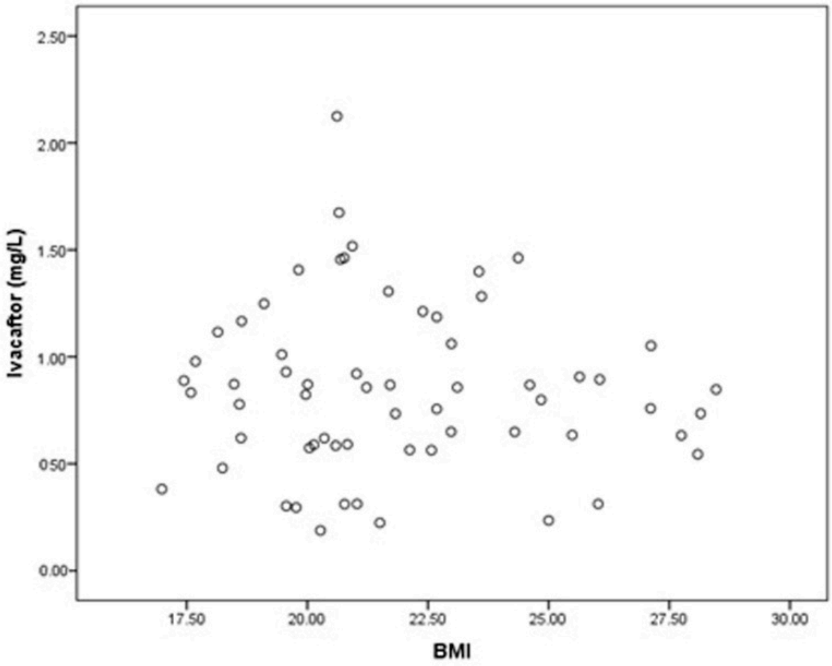

Supplement: Supplementary file 1 [file biomedicines-11-00628-s001.zip › biomedicines-2203000-supplementary.pdf]
